# Covariate selection strategies and estimands - a review of current practice of risk factor analysis from a causal perspective

**DOI:** 10.1186/s12874-025-02704-0

**Published:** 2025-11-19

**Authors:** Ragna Reinhammar, Ingeborg Waernbaum

**Affiliations:** Department of Statistics, Uppsala University, Kyrkogårdsgatan 10, Uppsala, 753 12 Sweden

**Keywords:** Causal language, Causal model, Model misspecification, Observational studies, Regression analysis, Selection algorithms

## Abstract

In applied epidemiological studies, the term “risk factor” is used synonymously with both predictors and causal factors for disease, although causal aims of explanatory analyses are rarely stated explicitly. Consequently, the concepts of explaining and predicting are conflated in many risk factor analyses, where data-driven variable selection, not suitable for causal inference, is common. We review the current practice of evaluating risk factors with regression analysis in three medical journals, focusing on the definition of risk factors, covariate selection strategies, and interpretation of regression coefficients. In simulations, we compare common practices to a causal approach using a data learner that mimics data from a study investigating risk factors for COVID-19 severity among patients with Multiple Sclerosis. The implied meaning of the term’ risk factor’ varies across the reviewed articles, but many authors implicitly give a causal definition of the term. In the articles, logistic regression is the most frequently used model, from which the coefficients are taken as effect estimates of the risk factors under study. We identify three common covariate selection strategies: i) adjusting for a pre-specified set, ii) stepwise selection, and iii) univariable pre-filtering. The simulation study illustrates the limitations of the current practice compared to the estimation of a marginal causal odds ratio by highlighting the difference between the conditional odds ratio and the marginal odds ratio. When considering risk factors as causal factors, the effect of interest should be clearly defined, and variables should be selected based on the underlying causal structure.

**Supplementary Information:**

The online version contains supplementary material available at 10.1186/s12874-025-02704-0.

## Introduction

Much modern epidemiological research aims to explain disease variation by identifying risk factors. Although the term “risk factor” is ambiguous in empirical research, it is commonly used to refer to variables associated with an increased probability of disease occurrence [1, 2]. However, the term ’risk factor’ is also used causally, in the context of identifying and designing preventive measures for disease [3, 4]. When the research involves causal assumptions about the underlying mechanisms, describing risk factors using associational language creates ambiguity regarding the research purpose, as well as misleading inferences [5–7]. A typical example is describing the research purpose in associational terms, while recommending interventions on the identified risk factors in the discussion or conclusion [8].

In observational studies of risk factors, a common statistical tool is regression analysis. However, if the purpose is to guide policy-making or public health interventions, i.e. to estimate a causal population effect, conventional regression analysis is susceptible to several fallacies. In standard regression analyses of risk factors, the regression coefficients and their confidence intervals are reported as measures of association [9]. However, there can be issues with comparability and interpretation of coefficients. Non-collapsibility of regression coefficients, in particular, has been a long-standing topic that has been repeatedly re-examined and discussed across various regression settings [10].

The covariates in a regression model often have the implicit or explicit purpose of adjusting for confounding. Covariate selection may be based on subject matter knowledge, although data availability and data-driven methods are common. Examples of data-driven covariate selection strategies are univariable pre-filtering, stepwise selection, and change-in-estimate methods, or a combination thereof [11–13]. However, these types of strategies are not developed to select sets that are sufficient for adjusting for confounding [14]. Furthermore, regression analysis relies on the assumption of a true, often parametric, model of the outcome, as discussed by Shmueli [5] and Breiman [15]. The fitted parametric model is likely a misspecification of the data-generating process, resulting in bias that does not vanish with an increased sample size.

In contrast, the causal inference literature emphasises causal estimands, usually describing marginal effects, either in the total population or in different subgroups [16, 17]. A causal estimand is non-parametric and does not depend on the specification of the regression model. When parametric regression models are used in this context, they are explicitly defined as working nuisance models, and approaches for bias reduction and robustness to model misspecification have been developed. See, e.g., Vansteelandt et al. [18].

In addition, the causal inference literature offers criteria for selecting adjustment sets sufficient for identifying the causal estimand of interest [19]. Covariates are selected based on a causal model summarised in a Directed Acyclic Graph (DAG), which takes the causal structure among variables into account. A causal model for the variables in the data at hand is rarely controversial but rather displays that, for example, baseline characteristics such as age and sex are not caused by variables defined later in life [20].

In this paper, we study how the term “risk factor” is currently used in medical research, as well as how regression analysis and covariate selection are applied in risk factor studies. This is done through a literature review, in which we investigate study objectives, causal language, and covariate selection strategies in three influential medical journals. We describe the journals’ guidelines and instructions to authors regarding regression analysis, covariate selection, and causal language. Then, we contrast conventional interpretations of logistic regression coefficients with approaches that recover marginal effects, including regression-based and doubly robust estimators, in a simulation study. One of the articles in the review, Louapre et al. [21], is used for inspiration for the construction of a data learner: mscovid_sim.

The paper is organised as follows. The Literature review section describes and presents the results of the literature review. In the Potential outcomes and causal estimands section model and theory defining the causal framework and estimands are given, and the Data learner: mscovid_sim section describes the data learner: mscovid_sim. A Monte Carlo simulation study using the data learner is presented in the Monte Carlo simulation study section. The results are discussed in the Discussion section. The paper is concluded with the key findings and recommendations for practitioners in the Conclusions section.

## Literature review

For the literature review, papers were sought and gathered from PubMed, using the keywords “risk factor(s)” and “regression”. Four major medical journals were selected: the Lancet, the British Medical Journal (BMJ), the Journal of the American Medical Association (JAMA), and the New England Journal of Medicine (NEJM), as well as their associated journals. The exact search string is presented in Appendix A. The articles were filtered on study type and publication year. Only observational studies published online and available as free full text during 2020 and 2021 were eligible for inclusion.

**Fig. 1:**
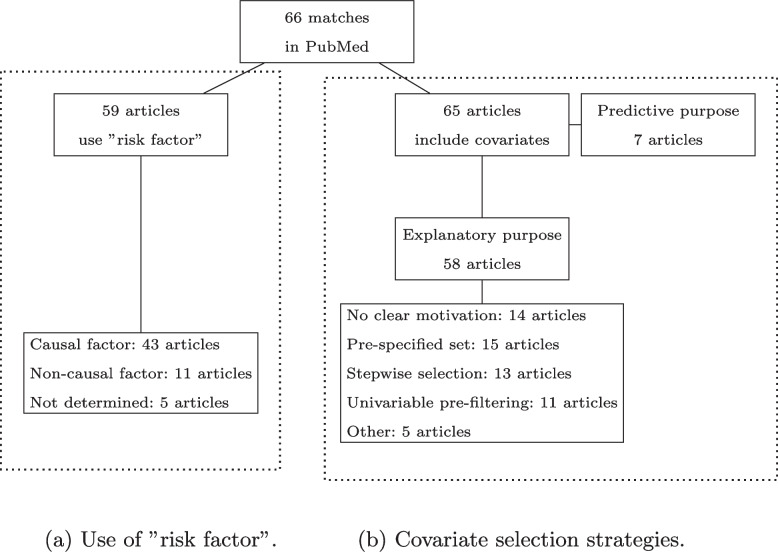
Flow diagram displaying article selection and main findings

The PubMed search was executed on the 25th of July 2022 and gave 66 matches. Figure 1 displays the selection process and the main results of the literature review. The full list of articles and the categorisation is available on Github, and is linked in Appendix A. There were 16 articles published in the Lancet, 33 in the BMJ, 17 in JAMA, and no article was published in NEJM. One author assessed the articles. The categorisation of causal language and covariate selection strategies was determined inductively by both authors based on the findings.

The included articles were evaluated based on how the term “risk factor” was used, whether it was causal, associative, or predictive. The use was judged by reading the parts of the text that included the term. Articles that made a causal definition of risk factor were classified as “Causal factor”. Articles in which the language regarding risk factors was consistently associational or predictive were classified as “Non-causal factor”. Articles that did not use the term in such a way that it was clear if they were considered causal factors, predictors, or associated factors were classified as “Not determined”.

Furthermore, all included articles were assessed to determine whether their purpose was explanatory or predictive. The purpose was judged by reading the objective, abstract and introduction. If the purpose was unclear, the full text was considered. Articles that aimed to predict an outcome or develop a prognostic model were categorised as predictive. Articles were categorised as explanatory if they aimed to evaluate an association between a treatment and an outcome, or to evaluate independent risk factors of a disease. Articles with a mixed purpose were categorised as explanatory. If the article focused on the estimated coefficients rather than prediction accuracy, it was categorised as explanatory.

The articles with an explanatory purpose were categorised based on their covariate selection strategy. The covariate selection strategy was determined by reading the section on statistical methods. If the description of statistical methods did not mention the covariate selection strategy, the results section was examined. If data-driven and subject matter knowledge were combined, the selection strategy was categorised based on the data-driven approach.

There were 58 articles with an explanatory purpose. Out of these, 32 used logistic regression for their statistical analysis. All of these reported odds ratios as measures of association. Many reported both odds ratios from multiple regression and unadjusted odds ratios. Other common regression models were Poisson regression and Cox proportional hazards regression. Seven articles had a predictive purpose, and they were therefore excluded from further assessment on the covariate selection strategy.

### Use of “risk factor” and causal language

Out of the 66 included articles, 59 used the term “risk factor” in the text. The common usages of the term “risk factor” are summarised in Table 1. Medical journals often advise against using causal language in observational studies, and therefore authors usually refrain from using explicit causal language, see the discussion in [6]. For example, JAMA only allow causal language when the study is a randomised controlled trial [22]. NEJM do not allow causal language in observational studies [23]. Regardless of these guidelines, some authors made implicit causal definitions of “risk factor” which we divide into three types of definitions below.

**Table 1:**
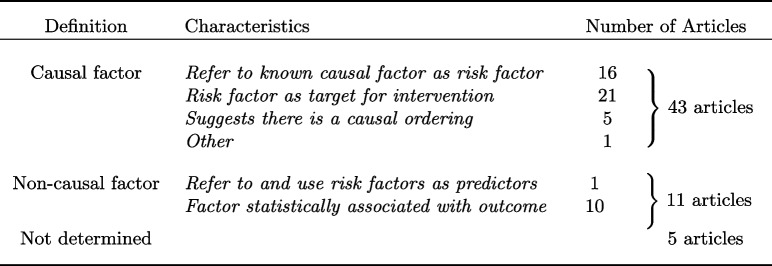
Definitions of risk factors and use of causal language in the reviewed articles

The first type of causal definition was to refer to a known causal factor as a risk factor. For example, Mei et al. [24] assess the risk factors for early childhood caries in Wenzhou, China. One of the studied risk factors is consumption of sugary snacks and drinks. Consuming sugar is known to cause caries, making consumption of sugary snacks and drinks a causal risk factor.

The second type of implicit causal definition was to discuss risk factors as targets for interventions or actions. For example, Louapre et al. [21] studied risk factors of COVID-19 severity among patients with Multiple Sclerosis. One of the studied risk factors is disease-modifying therapy. The authors could not find a statistically significant association between disease-modifying therapy and COVID-19 severity, and therefore conclude that their results reinforce the recommendation of continuing disease-modifying therapies during the pandemic.

The last type of implicit causal definition is to suggest a causal ordering of factors. Shinohara et al. [25] write that elective cesarean section before the onset of uterine contractions is a risk factor of transient tachypnoea of the newborn (TTN). They continue to state that since the rate of elective cesarean section will increase, the number of newborns with TTN will inevitably increase. This suggests that cesarean section before the onset of uterine contractions has a causal effect on the risk of TTN.

### Covariate selection strategies

All four journals have instructions for authors on their respective websites, including guidelines on statistical reporting in observational studies. The Lancet requires observational studies to report in accordance with the STROBE statement, a checklist of 22 items on how to clearly report observational studies [26, 27]. The BMJ also refers to the STROBE statement and appropriate extensions, as well as the SAMPL guidelines [28]. The SAMPL guidelines focus on reporting the most common statistical analyses [29]. The NEJM requires that observational studies describe how measured and unmeasured confounding was managed. Furthermore, studies that report on a treatment effect should illustrate the distribution of confounders by treatment level. In addition, authors are encouraged to quantify sensitivity to potential bias from unmeasured confounding [23]. JAMA requires authors to adhere to guidelines published on the Equator Network’s website, which include the STROBE statement and its extensions, as well as guidelines for other types of studies [22, 30]. Authors are also referred to *Reporting statistical information in medical journal articles* [31]. The article discusses what values to include and which to omit, how to do power calculations, and some writing recommendations. Furthermore, JAMAs instructions request authors to describe the scientific rationale for including variables in a regression model, whether it is clinical, statistical, or other [22].

Three common covariate selection strategies were identified, although many did not clearly motivate the choice of covariates. Table 2 gives a detailed description of the findings. The most common strategy did not report any data-driven covariate selection. Instead, articles in this category included all covariates in a pre-specified set. The motivation for the covariates in the set varied. For example, some authors motivated the included covariates by previous research. The second most common strategy was stepwise selection, either from a pre-specified set of covariates (seven articles) or from a set selected through univariable pre-filtering (six articles). Some authors mentioned using the Akaike Information Criteria (AIC) or *p*-values for the inclusion or exclusion of covariates, while others did not mention which criteria they used. The third most common variable selection strategy was univariable pre-filtering. In univariable pre-filtering, all covariates that are significant in “univariable” analyses are selected. Examples of univariable analyses are simple regression or hypothesis tests of difference in means between outcome groups. In total, 11 articles implemented some type of univariable pre-filtering.

**Table 2:**
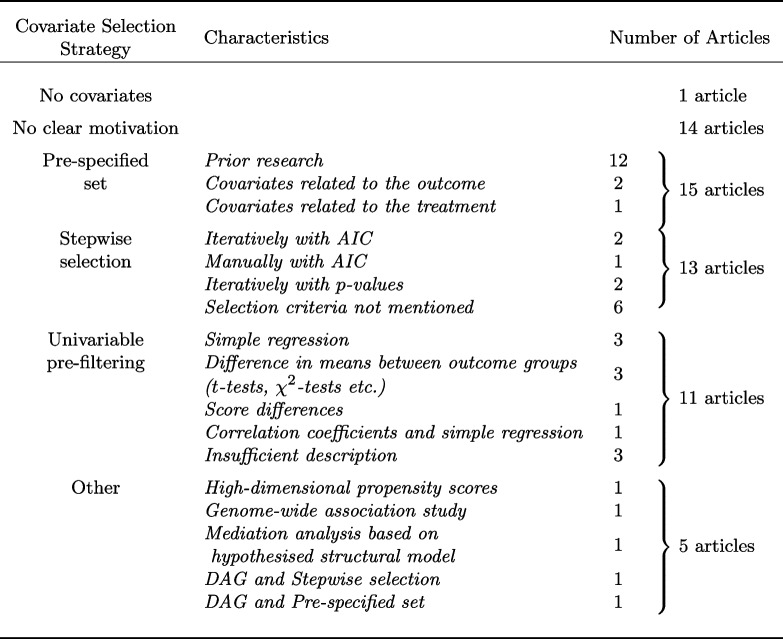
Description of covariate selection strategies identified in the literature review

Five articles did not naturally fall into any of the three categories and were categorised as Other. One used high-dimensional propensity scores, one was a genome-wide association study, one was a mediation analysis that included a hypothesised structural model, one did stepwise inclusion of covariates but included a causal model in the form of a DAG, and the fifth adjusted for a pre-specified set but also included a DAG. The last three articles were the only articles that presented a graphical description of the hypothesised underlying causal structure.

## Potential outcomes and causal estimands

In the sequel, we describe estimands and estimation approaches when the objective of the analysis is to estimate the causal effect of a risk factor on a binary outcome, for example, a disease. We assume a sample of *n* independent and identically distributed individuals $i=1,\ldots ,n$. We assume a multi-valued risk factor, or treatment, $T_i\in \mathbb {T}={1,\ldots ,L}$, where the standard case for binary treatments $L=2$ is included. We use $T_i=t$ if the individual received treatment level *t*, and $T_i=t'$ if the individual received treatment level $t'$. We let $\varvec{X}$ denote a set of variables, referred to as covariates, which includes the pool of possible risk factors for the binary outcome $Y$.

The description of estimators and necessary assumptions is based on the potential outcome framework [32, 33]. We define *L* indicator variables $D_{i}(t) = \mathbb{I}\{ T_i = t\}$ taking the value 1 for $T_i=t$. The potential outcomes under treatment level $t$ and $t'$ are denoted $Y(t)$ and $Y(t')$, respectively. The observed outcome is denoted *Y* and throughout, we assume consistency, i.e. that the observed outcome is the potential outcome under the treatment received: $Y=Y(t)$ if $T=t$. The vector of observed data is thus $(Y_i,T_i,\varvec{X}_i)$. We denote the probability of being treated with treatment *t* conditional on the covariates, the *propensity score*, by $e_t(\varvec{x}_i)=P(T_i=t|\varvec{X}=\varvec{x})$. In the sequel, we drop the subscript *i* for the random variables when not needed.

The marginal causal odds ratio (MCOR) compares two treatment levels $t$ and $t'$:

1 $$\begin{aligned} \theta _{t|t'} = \frac{p(Y(t) = 1)/p(Y(t)=0)}{p(Y(t') = 1)/p(Y(t')=0)} \end{aligned}$$

which contrasts two hypothetical worlds: everyone receiving treatment *t*, versus everyone receiving treatment $t'$.

The conditional causal odds ratio (CCOR) $\theta _{t|t'}(\varvec{x})$ instead compares odds of units with characteristics $\varvec{X}=\varvec{x}$:

2 $$\begin{aligned}\theta _{t|t'}(x)\nonumber = \frac{p(Y(t) = 1 \ | \ T = t, \varvec{X}=\varvec{x})/p(Y(t) = 0 \ | \ T = t,\varvec{X}=\varvec{x}) }{p(Y(t') = 1 \ | \ T = t',\varvec{X}=\varvec{x})/p(Y(t')= 0 \ | \ T = t', \varvec{X}=\varvec{x})}. \end{aligned}$$

In observational studies, when the treatment is not assigned at random, identification strategies for causal analysis often make use of an assumption of no unmeasured confounding and positivity given below:

**A1.** (No unmeasured confounding) *If* $\varvec{X}$ *is sufficient for confounding control, then we have that the treatment, t, and the potential outcomes are independent, given the covariates:*

$$\begin{aligned} D(t) \ \perp \!\!\! \perp \ Y(t) \ | \ \varvec{X}, \ \forall t \in \mathbb {T}. \end{aligned}$$

**A2.** (Positivity) *For any given values of* $\varvec{X}$*, the probability to receive any level of the treatment T is positive:*

$$\begin{aligned} 0< p(T= t \ | \ \varvec{X} = \varvec{x}) < 1, \forall t \in \mathbb {T} \text { and } \forall \varvec{x} \in \mathbb {X}. \end{aligned}$$

We also formulate an assumption of pre-treatment covariates:

**A3.** (Pre-treatment covariates) *The covariates in* $\varvec{X}$ *are measured prior to treatment.*

A set $\varvec{X}^*\subseteq \varvec{X}$ satisfying assumptions **A1**-**A3** is a *sufficient adjustment set* [14], i.e. sufficient to infer causal effects from a non-randomised study. A sufficient adjustment set includes all confounders, but excludes mediators (on the causal pathway) and colliders (influenced by both treatment and outcome).

Under assumption **A1**-**A3**, the CCOR is identified since

3 $$\begin{aligned} \theta _{t|t'}(\varvec{x})\nonumber & = \frac{p(Y=1|T=t, \varvec{X}=\varvec{x})/p(Y=0|T=t, \varvec{X}=\varvec{x})}{p(Y=1|T=t', \varvec{X}=\varvec{x})/p(Y=0|T=t', \varvec{X}=\varvec{x})}\nonumber \\ & =\frac{p(Y(t)=1|T=t, \varvec{X}=\varvec{x})/p(Y(t)=0|T=t, \varvec{X}=\varvec{x})}{p(Y(t')=1|T=t', \varvec{X}=\varvec{x})/p(Y(t')=0|T=t', \varvec{X}=\varvec{x})}. \end{aligned}$$

Similarly the MCOR is identified under assumptions **A1**-**A3** since by taking expectations on each of the components in (3), $E[p(Y =1 | T=t,\varvec{X}=\varvec{x})]=p(Y(t)=1)$ and $E[p(Y =0 | T=t,\varvec{X}=\varvec{x})]=p(Y(t)=0)$.

There are multiple criteria to select sufficient adjustment sets based on Directed Acyclic Graphs, illustrating an assumed underlying causal structure. When the full DAG is known, sufficient adjustment sets can be selected with the back-door criterion [19]. We recommend the six-step summary by Shrier and Platt [34] that focuses on analysing medical data. A recent comprehensible tutorial on directed acyclic graphs aimed for clinical epidemiologists is available [35]. If the causal structure is partially known, the disjunctive cause criterion, including variables whose elements are either causes of *T* or of *Y* or of both, can also be used for selection [36]. Additionally, if **A1**-**A3** hold for the full set of covariates $\varvec{X}$, the selection of a subset that is a sufficient adjustment set can be made with algorithms using the observed data [37]. The algorithms of [37] are implemented in the R package CovSel. As an example, the set of predictors of the treatment or outcome is sufficient to adjust for confounding. In that case, the set of predictors of the outcome may be the more efficient choice [14]. To assess the robustness to possible unmeasured confounding, sensitivity analysis can be performed. An accessible approach for the data analyst is to use the E-value that comes with a simple interpretation [38]. An R-package Evalue is available to implement the analysis.

### Estimation

Consider the logistic regression model

4 $$\begin{aligned} \text {logit}\left[ p(Y=1| T=t, \varvec{X}=\textbf{x}) \right] = \alpha +\beta \cdot t+\varvec{\gamma '}\varvec{x}, \end{aligned}$$

with $\hat{\theta }^{reg}_{t|t'} = \exp \{\hat{\beta } \}$ as an estimator for the CCOR. The estimator $\hat{\theta }^{reg}_{t|t'}$ is often referred to as an adjusted odds ratio, or a multivariable or multivariate-adjusted odds ratio. See e.g. Eng et al. [39] for an example from the literature review.

Under assumptions **A1-A3** and assuming the model in (4) is correctly specified, $\hat{\theta }^{reg}_{t|t'}$ is a consistent estimator of the CCOR. However, the value of the CCOR depends on which other variables are included in the covariate set $\varvec{X}$. This means that two observational studies of a disease, using different sufficient adjustment sets, will not produce comparable estimates of $\theta _{t|t'}(\varvec{x})$. When comparing two logistic regression models, where we adjust for different sets of covariates, the differences seen in $\hat{\theta }^{reg}_{t|t'}$ between models are due to a non-collapsibility effect if the variables in the models are predictors of the outcome [40]. This makes it difficult to compare or validate an excess odds. Therefore, direct interpretation of a logistic regression coefficient as an adjusted odds ratio is advised against, see for example the discussion in Daniel et al. [41].

Besides violations of model assumptions in (4), the assumption **A3** is not fulfilled if $\varvec{X}$ includes mediators. In this case, the regression coefficient may not represent the total effect of treatment, but the controlled direct effect, which is the part of the effect that is not mediated through any mediators. This may very well be the case when a set of covariates is entered in a multiple regression model, and all regression coefficients for variables in $\varvec{X}$ are evaluated as effects of risk factors, without taking the underlying causal structure into account (known as the Table 2 fallacy) [42]. See [43] for an example from the review. Greenland [44] further investigated collider stratification bias, and potential sizes of confounding and collider stratification biases are discussed and compared.

However, logistic regression may still be useful for estimating population effects. In the current paper, we discuss two other estimators that may be derived with the same type of outcome regression model. One alternative is to marginalise over the distribution of a sufficient adjustment set. Then the MCOR can be identified from the conditional probabilities by taking the expectation $E[p(Y =1 | T=t, \varvec{X})] = p(Y(t)=1)$. To be more general, we define *L* outcome models

5 $$\begin{aligned} P(Y(t)=1|\varvec{X}=\varvec{x})=g_t(\varvec{x}). \end{aligned}$$

for $t=1,\ldots , L$. The models $g_t()$ can be parametric, e.g. logit or probit models, or nonparametric, for example, classification trees. An estimator of the MCOR can be obtained by taking the empirical mean of the predicted probabilities in (5):

6 $$\begin{aligned} \hat{E}[p(Y =1 | T=t,\varvec{X}=\varvec{x}]=\frac{1}{n}\sum _{i=1}^{n}\hat{g}_t(\varvec{x}_i) \end{aligned}$$

If parametric models are used, the predicted probabilities $\hat{g}_t(\varvec{x}_i)$ are obtained by first fitting the model for each treatment group separately and then subsequently predicting probabilities for the whole sample of *n* units. We construct the resulting Regression Imputation (RI) estimator of the MCOR, $\hat{\theta }^{ri}_{t|t'}$:

7 $$\begin{aligned} \hat{\theta }^{ri}_{t|t'} = \frac{\sum _{i=1}^n \hat{g}_t(\varvec{x}_i) \big /\sum _{i=1}^n \left( 1- \hat{g}_t(\varvec{x}_i) \right) }{\sum _{i=1}^n \hat{g}_{t'}(\varvec{x}_i) \big /\sum _{i=1}^n \left( 1- \hat{g}_{t'}(\varvec{x}_i) \right) }. \end{aligned}$$

Properties of regression imputation estimators for the MCOR can be obtained under regularity conditions, see e.g. the proposal by Zhang for logistic regression [45]. The regression imputation estimator is also known as the g-computation estimator, or the standardisation estimator [46].

An alternative estimator of the MCOR is based on the Augmented Inverse Probability Weighting (AIPW) estimator proposed by Robins and Rotnitzky [47], which combines the properties of the Inverse Probability Weighting (IPW) estimator and a regression-based estimator. IPW uses the estimated propensity score, $\hat{e}_t(x_i)$, as weights, and when correctly specified, it can reduce bias and increase efficiency. The IPW estimator is defined as:

8 $$\begin{aligned} & \hat{\theta }^{ipw}_{t|t'}\nonumber = \frac{\frac{1}{n}\sum \limits _{i=1}^{n} \left[ \frac{y_i D_{i}(t)}{\hat{e}_t(\varvec{x}_i)} - \frac{D_{i}(t) - \hat{e}_t(\varvec{x}_i)}{\hat{e}_t(\varvec{x}_i)}\right] \big /\left[ 1- \frac{1}{n}\sum \limits _{i=1}^{n} \left[ \frac{y_i D_{i}(t)}{\hat{e}_t(\varvec{x}_i)} - \frac{D_{i}(t) - \hat{e}_t(\varvec{x}_i)}{\hat{e}_t(\varvec{x}_i)}\right] \right] }{\frac{1}{n}\sum \limits _{i=1}^{n} \left[ \frac{y_i D_{i}(t')}{\hat{e}_t'( \varvec{x})} - \frac{D_{i}(t') - \hat{e}_t'( \varvec{x})}{\hat{e}_t'( \varvec{x})}\right] \big /\left[ 1- \frac{1}{n}\sum \limits _{i=1}^{n} \left[ \frac{y_i D_{i}(t')}{\hat{e}_t'( \varvec{x})} - \frac{D_{i}(t') - \hat{e}_t'( \varvec{x})}{\hat{e}_t'( \varvec{x})}\right] \right] }. \end{aligned}$$

The AIPW estimator for the MCOR is then defined as:

9 $$\begin{aligned} & \hat{\theta }^{aipw}_{t|t'}\nonumber = \frac{\frac{1}{n}\sum \limits _{i=1}^{n} \left[ \frac{y_i D_{i}(t)}{\hat{e}_t(\varvec{x}_i)} - \frac{D_{i}(t) - \hat{e}_t(\varvec{x}_i)}{\hat{e}_t(\varvec{x}_i)}\hat{g}_t(\varvec{x}_i)\right] \big /\left[ 1- \frac{1}{n}\sum \limits _{i=1}^{n} \left[ \frac{y_i D_{i}(t)}{\hat{e}_t(\varvec{x}_i)} - \frac{D_{i}(t) - \hat{e}_t(\varvec{x}_i)}{\hat{e}_t(\varvec{x}_i)}\hat{g}_t(\varvec{x}_i)\right] \right] }{\frac{1}{n}\sum \limits _{i=1}^{n} \left[ \frac{y_i D_{i}(t')}{\hat{e}_t'( \varvec{x})} - \frac{D_{i}(t') - \hat{e}_t'( \varvec{x})}{\hat{e}_t'( \varvec{x})}\hat{g}_{t'}(\varvec{x}_i)\right] \big /\left[ 1- \frac{1}{n}\sum \limits _{i=1}^{n} \left[ \frac{y_i D_{i}(t')}{\hat{e}_t'( \varvec{x})} - \frac{D_{i}(t') - \hat{e}_t'( \varvec{x})}{\hat{e}_t'( \varvec{x})}\hat{g}_{t'}(\varvec{x}_i)\right] \right] }. \end{aligned}$$

The AIPW estimator includes estimated propensity scores $\hat{e}_t(\varvec{x})$ and $\hat{e}_t'(\varvec{x})$ for the contrasts $t, t'$ commonly estimated with either a parametric logistic regression for the binary treatment case or with multinomial logistic regression model in the multi-valued case. However, neither the regression imputation estimator, $\hat{\theta }^{ri}_{t|t'}$, nor the IPW estimator, $\hat{\theta }^{ipw}_{t|t'}$, and the AIPW estimator, $\hat{\theta }^{aipw}_{t|t'}$, are restricted to logistic regression. They can be estimated using other methods, either parametric or nonparametric. In the Monte Carlo simulation study, we describe several implementations of the estimators. Because the IPW estimator does not include any outcome model, it is not discussed further in the current paper.

## Data learner: mscovid_sim

To compare the methods identified in the Literature review to the marginal estimators of causal estimands described in the Estimation section, we introduce a data learner mscovid_sim. The data learner is implemented in R and mimics the data in Louapre et al. [21]. In the original article, the authors studied risk factors for COVID-19 severity among Multiple Sclerosis (MS) patients.

### Causal graph and data generating process

**Fig. 2:**
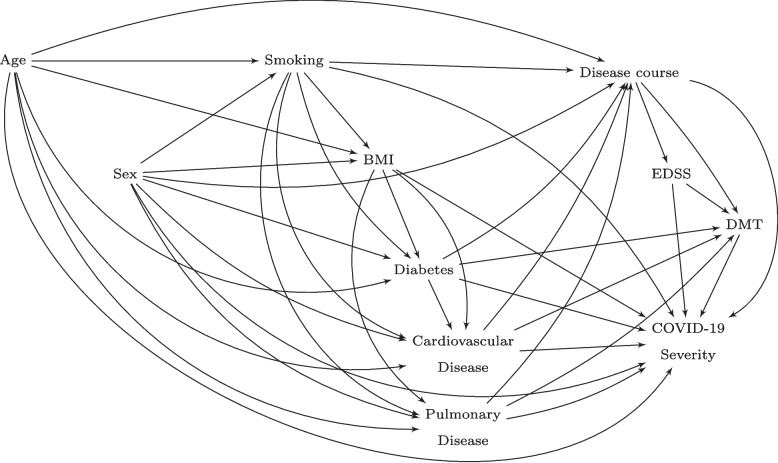
DAG displaying the data-generating model in the data learner. EDSS, Expanded Disability Status Scale. DMT, disease-modifying therapy. The outcome variable is COVID-19 severity. The treatment variable is DMT in Scenario I and Smoking in Scenario II

The hypothesised causal model that we used to generate the data in mscovid_sim is displayed in the DAG of Fig. 2. In the original article by Louapre et al., the risk factors under study were Age, Sex, Smoking status, BMI, Diabetes status, presence of cardiovascular disease, presence of pulmonary disease, Disease Course (type of MS), Expanded Disability Status Scale (EDSS), Disease-modifying Treatment (DMT). EDSS is a scale for evaluating disability in patients with MS [48]. DMTs are treatments for MS. The binary outcome, Severe COVID-19, is defined as having a severity score of 3 or more on a 7-point scale. Scores 3 to 7 include all outcomes from being hospitalised without requiring supplemental oxygen, to death [21].

The simulated data was generated such that sample means and proportions in the generated samples are approximately equal to those in Louapre et al. The approximated sample quantities are listed in the column “Target” in Table B2 in Appendix B. The data-generating process of the covariates and treatments is described in detail in Table B3. The data learner is available on Github, see link in Appendix B.

Two scenarios, I and II, were studied. In Scenario I, the treatment is Disease-Modifying Therapy (DMT). In Scenario II, the treatment is Smoking. In the first scenario, the set of all covariates is a sufficient adjustment set. Another, smaller, sufficient adjustment set includes the parents of DMT, which are EDSS, Disease Course, and Comorbidities (Diabetes, Cardiovascular Disease, Pulmonary Disease). In Scenario II, the full set of covariates is not a sufficient adjustment set since it includes mediators, i.e., **A3** does not hold. Instead, the set consisting of the parents of Smoking, Age and Sex, makes a sufficient adjustment set. This is both the minimal and maximal sufficient set.

Following the original data, there are 12 underlying DMTs which were grouped into four categories: No treatment (0), No risk (1), Low risk (2), and Moderate to high risk (3). The corresponding marginal probabilities were set to $p(Y(0) = 1) = 0.19$, $p(Y(1) = 1) = 0.19$, $p(Y(2) = 1) = 0.20$, and $p(Y(3) = 1) = 0.25$, yielding the following MCORs

$$\begin{aligned} \text {DMT}: \theta _{1|0} = 1, \ \theta _{2|0} = 1.066, \text { and } \theta _{3|0} = 1.421. \end{aligned}$$

Data was generated such that the different types of DMTs within each risk level are equal in terms of the increased risk of severe COVID-19. Here, one patient’s DMT does not affect another patient’s risk of severe COVID-19, meaning that the stable unit treatment value assumption (SUTVA) is fulfilled.

Smoking is binary, with 0 representing non-smoker and 1 representing smoker. The marginal probabilities were set to $p(Y(0)=1) = 0.205$ and $p(Y(1) = 1) = 0.24$, yielding the MCOR

$$\begin{aligned} \text {SMOKING}: \theta _{1|0} = 1.225. \end{aligned}$$

Furthermore, data was generated such that the condition of SUTVA is met.

In each scenario, the outcome, COVID-19 Severity, was generated in three ways, referred to as Outcome models A, B and C. Outcome model A is a logistic model with only linear terms. Outcome model B is a logistic model with interaction between the treatment and the covariates. Outcome model C is a probit model with non-linear terms and interactions between covariates, as well as interactions between the treatment and covariates. The outcome generating models are described in detail in Table B4 in Appendix B. The observed outcome is the potential outcome corresponding to the treatment level received.

### Estimation

CCORs $\theta _{t|t'}(x)$ were estimated with $\hat{\theta }^{reg}_{t|t'}$ conditioning on different covariate sets of the covariate selection strategies identified in the Literature review: all covariates (ALL), stepwise selection (SS), and univariable pre-filtering (UPF). When implementing the selection strategies, the true parameter $\theta _{t|t'}(\varvec{x})$ differs both between approaches but also between replications, hence emphasising the aforementioned difficulties of comparison and interpretation. The MCOR was estimated with both $\hat{\theta }^{ri}_{t|t'}$ and $\hat{\theta }^{aipw}_{t|t'}$ with a sufficient adjustment set in the outcome model. The estimator $\hat{\theta }^{aipw}_{t|t'}$ (AIPW) was used both under a correctly specified and a misspecified propensity score model. Under the misspecified propensity score, the estimator is denoted $\hat{\theta }^{aipw*}_{t|t'}$ (AIPW^*^). In addition, both $\hat{\theta }^{ri}_{t|t'}$ and $\hat{\theta }^{aipw}_{t|t'}$ were estimated non-parametrically (npRI, npAIPW). The estimators were compared to the unadjusted (UA) odds ratio as well. Table 3 provides a summary of estimators and notation.

**Table 3:** Summary of the estimators and covariate selection methods in the simulation study

Notation	Estimator	Covariates adjusted for	Model
UA	Crude odds ratio	None	-
*Target estimand: CCOR*			
ALL	$\hat{\theta }^{reg}_{t|t'}$	Full set	Logistic regression
SS	$\hat{\theta }^{reg}_{t|t'}$	Stepwise selection from the full set	Logistic regression
UPF	$\hat{\theta }^{reg}_{t|t'}$	Univariable pre-filtering from the full set	Logistic regression
*Target estimand: MCOR*			
RI	$\hat{\theta }^{ri}_{t|t'}$	Full set/parents of treatment	Logistic regression
AIPW	$\hat{\theta }^{aipw}_{t|t'}$	Full set/parents of treatment	(Multinomial) Logistic regression
AIPW^*^	$\hat{\theta }^{aipw}_{t|t'}$	Full set/parents of treatment	(Multinomial) Logistic regression
npRI	$\hat{\theta }^{ri}_{t|t'}$	Full set/parents of treatment	Random Forest
npAIPW	$\hat{\theta }^{aipw}_{t|t'}$	Full set/parents of treatment	Random Forest

Abbreviations are listed at the beginning of the paper

AIPW^*^ *uses an incorrectly specified propensity score model. np stands for non-parametric*

We implemented the estimators in R. The estimator $\hat{\theta }^{aipw}_{t|t'}$ was implemented with the R package PSweight [49]. The package supports both binary and multi-valued treatments. In the nonparametric versions, the propensity scores and conditional outcome probabilities were estimated using random forests implemented with the R package ranger [50] using default values for parameter tuning.

The SS and UPF estimators may be subject to confounding in both scenarios, depending on which covariates were included in the adjustment set. The SS estimator implemented backward elimination and forward selection based on AIC. The UPF estimator adjusted for the covariates that are significant at the 5 % level in simple logistic regression.

All parametric estimators assume an incorrect model form specification under outcome models B and C. Under outcome model B, they were missing the treatment-covariate interaction. Under outcome model C, they were missing non-linear terms and interactions, and also applied the wrong link function.

## Monte Carlo simulation study

A Monte Carlo simulation study was conducted to evaluate the estimators in 3.1. The empirical distribution of the estimators is illustrated with box plots where the bias and Mean Squared Error (MSE) are portrayed with respect to the true MCOR. In the simulation, 1000 samples were drawn from the data learner. The sample size is 3470, ten times larger than in Louapre et al. [21]. Monte Carlo standard errors (MCSEs) are reported in Appendix D. In each scenario, the overlap in the first sample was assessed with density plots, which are displayed in Appendix C.

An expression for the asymptotic bias of the parametric marginal estimators is given in Appendix E. The asymptotic bias of the unadjusted odds ratio as well as of the ALL, RI, AIPW, and AIPW${}^{*}$ estimators was approximated numerically by estimating them in a sample of 1 million observations. The approximated bias is also reported in Appendix E.

### Results: scenario I

**Fig. 3:**
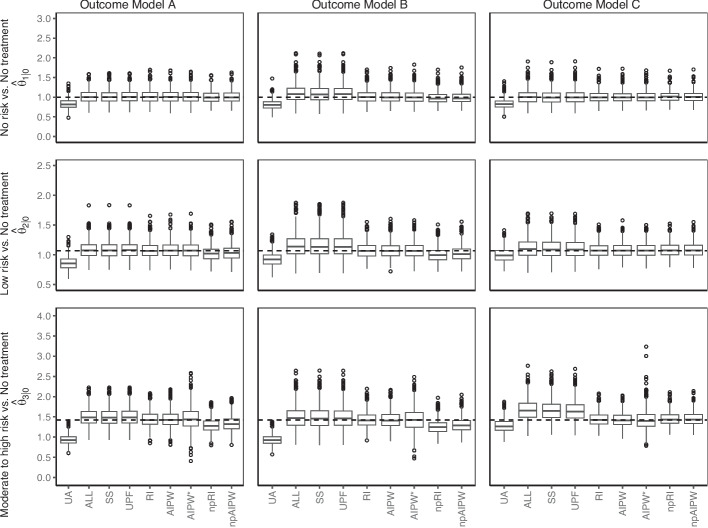
Data learner, scenario I, estimates of treatment effect of *DMT* on *COVID-19 severity*

The estimated odds ratios under each outcome model are displayed in Fig. 3 and the bias and MSE of the estimators are presented in Table 4. The unadjusted odds ratio consistently underestimated the odds ratios across all outcome models. It shows a large negative bias but did not have a larger variance than the other estimators. The conditional estimator $\hat{\theta }^{reg}_{t|t'}$ implemented with the covariate selection strategies ALL, SS and UPF were in general larger than the MCOR, except for $\hat{\theta }^{reg}_{1|0}$, and $\hat{\theta }^{reg}_{2|0}$ under outcome models A and C. In the other cases, the bias was especially large for $\hat{\theta }^{reg}_{3|0}$ for outcome model C. The MSE was also slightly larger in some cases, due to the conditional estimators having a larger variance than some of the others. The parametric regression imputation estimator performed well under all outcome models since the separate fitting of the models for the different treatments mitigated the missing interaction terms. The parametric AIPW estimators were relatively stable across contrasts and outcome models. However, the AIPW${}^{*}$ estimator was quite variable for the largest contrast $\theta _{3|0}$. Both AIPW estimators show small biases, but the AIPW${}^{*}$ estimator had a larger MSE for the last contrast.

**Table 4:** Bias and MSE of estimators in scenario I

	Bias^1^			MSE		
Outcome Model:	A	B	C	A	B	C
$$\theta _{1|0}$$						
UA	−0.17	−0.19	−0.17	0.04	0.05	0.04
ALL	0.02	0.10	0.01	0.02	0.06	0.03
SS	0.01	0.09	0.00	0.02	0.06	0.03
UPF	0.02	0.10	0.01	0.03	0.06	0.03
RI	0.02	0.01	0.00	0.02	0.03	0.02
AIPW	0.02	0.01	0.00	0.03	0.03	0.02
AIPW*	0.02	0.01	0.01	0.03	0.03	0.02
npRI	0.00	−0.02	0.01	0.02	0.02	0.02
npAIPW	0.01	−0.01	0.01	0.02	0.02	0.02
$$\theta _{2|0}$$						
UA	−0.20	−0.14	−0.07	0.05	0.03	0.02
ALL	0.02	0.09	0.04	0.02	0.04	0.03
SS	0.02	0.09	0.04	0.02	0.04	0.03
UPF	0.02	0.09	0.04	0.02	0.04	0.03
RI	0.01	0.01	0.01	0.02	0.02	0.01
AIPW	0.01	0.01	0.01	0.02	0.02	0.01
AIPW*	0.01	0.01	0.01	0.02	0.02	0.01
npRI	−0.04	−0.06	0.02	0.02	0.02	0.01
npAIPW	−0.03	−0.04	0.01	0.02	0.02	0.01
$$\theta _{3|0}$$						
UA	−0.48	−0.49	−0.14	0.25	0.25	0.05
ALL	0.08	0.07	0.26	0.05	0.08	0.14
SS	0.07	0.07	0.24	0.05	0.08	0.12
UPF	0.08	0.07	0.23	0.05	0.08	0.11
RI	0.02	0.01	0.01	0.04	0.04	0.03
AIPW	0.02	0.01	0.01	0.04	0.04	0.03
AIPW*	0.03	0.01	0.01	0.08	0.08	0.06
npRI	−0.13	−0.16	0.03	0.04	0.05	0.03
npAIPW	−0.08	−0.12	0.02	0.04	0.04	0.03

^*1*^Bias defined with respect to the MCOR. AIPW^*^ uses an incorrectly specified propensity score model. np stands for non-parametric

The performance of the nonparametric estimators varied. They, in particular, the nonparametric RI, tended to underestimate the contrasts not equal to 1, under outcome models A and B. When inspecting the estimated potential outcomes from the random forest, it appears as though the random forest model may tend to overestimate the probabilities under no treatment. Under treatments, low probabilities tend to be underestimated. When the predicted probability under no treatment (the reference level) is overestimated, and the predicted probability under treatment is underestimated, the estimated odds ratio is closer to 1. The nonparametric AIPW estimator exhibited a slightly better performance compared to the nonparametric RI estimator. This could be attributed to the inclusion of the propensity score, which was also estimated using a random forest algorithm, contributing to the improved performance of the estimator. That nonparametric algorithms can perform worse than parametric regression was pointed out by Naimi et al. [51]. Their results especially suggested that Machine Learning methods based on a single model, which is the case here, should be avoided.

The biases in Table 4 were close to the approximated asymptotic biases in Table E7. Here, the asymptotic bias illustrates that the bias of the unadjusted and the conditional estimators observed in the simulation does not vanish with a larger sample size. The Monte Carlo Standard Errors (MCSEs) of bias and MSE are presented in Table D5. Generally, the standard errors of the marginal estimators were smaller than those of the conditional estimators, except the misspecified parametric AIPW estimator (AIPW${}^{*}$), which had larger standard errors.

### Results: scenario II

**Fig. 4:**
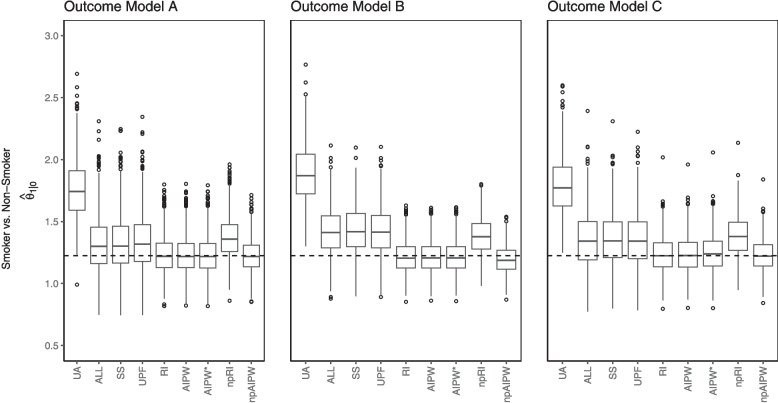
Data learner, scenario II, estimates of treatment effect of *smoking* on *COVID-19 severity*

The estimated odds ratios are displayed in Fig. 4 and the bias and MSE are presented in Table 5. The Monte Carlo standard errors of each measure are presented in Table D6. In this scenario, the unadjusted odds ratio overestimated the odds ratio, as do the conditional estimators. The parametric marginal estimators performed rather well. The nonparametric RI estimator overestimated the odds ratio. The random forest model struggled to predict the lower and higher outcome probabilities, particularly under no treatment. Under no treatment, especially, it failed to predict the larger probabilities, hence the overestimation of the odds ratio. The nonparametric AIPW estimator was better than the nonparametric RI estimator. Again, it appears that the propensity scores contribute to the estimation of the odds ratio, even though there was poor overlap for the larger propensity scores, particularly under the correct and non-parametric propensity score model. The overlap is displayed in Figs. C4 to C6.

**Table 5:** Bias and MSE of estimators in scenario II

	Bias^1^			MSE		
Outcome Model:	A	B	C	A	B	C
$$\theta _{1|0}$$						
UA	0.54	0.66	0.56	0.34	0.49	0.37
ALL	0.09	0.20	0.13	0.06	0.07	0.07
SS	0.10	0.21	0.14	0.06	0.08	0.07
UPF	0.11	0.20	0.13	0.06	0.08	0.06
RI	0.01	−0.01	0.01	0.02	0.02	0.02
AIPW	0.01	−0.01	0.01	0.02	0.02	0.02
AIPW*	0.01	−0.01	0.02	0.02	0.02	0.02
npRI	0.15	0.16	0.16	0.05	0.05	0.05
npAIPW	0.00	−0.03	0.00	0.02	0.01	0.02
^1^Bias defined with respect to the MCOR. AIPW^∗^ uses an incorrectly specified propensity score model. np stands for non-parametric						

The unadjusted and the conditional estimators had larger MSE than the marginal estimators. The marginal estimators had the smallest MSE of all, particularly the nonparametric AIPW estimator, as it showed both small bias and small variance.

As in the first scenario, the asymptotic bias was approximated (Table E8). Here, the asymptotic bias of the unadjusted estimator UA was the largest, illustrating the bias due to confounding. The discrepancy of the conditional estimator (ALL) was also large, but it instead demonstrates the difference between the CCOR and MCOR. The parametric RI and AIPW estimators showed consistently small, and even zero, bias.

## Discussion

This article gives a review of the current practice of evaluating risk factors with regression in three high-impact medical journals, focusing the definition of risk factors, covariate selection strategies, and interpretation of regression coefficients. Among the reviewed articles, many had an explanatory purpose. Within the review, various authors either implicitly defined risk factors causally, while others described them as predictors or factors statistically correlated with the outcome. Among the explanatory articles, three common covariate selection strategies were identified: i) including a pre-specified set, ii) stepwise selection, and iii) univariable pre-filtering. These three strategies have in common that the underlying causal structure of the covariate set is not considered when including or excluding covariates from the regression model. However, the review did identify three articles that used DAGs for variable selection. If the underlying causal structure is not considered, confounders may be wrongfully excluded, or mediators and colliders may be wrongly included. This would lead to biased estimators and misleading interpretations of the regression coefficients.

For the review, the categories of risk factor definitions and covariate selection strategies were determined inductively by both authors of this manuscript, although one author did the assessment. While it is possible that other reviewers would make different assessments, the goal of the current review was to categorise the articles without making subjective interpretations and to be fully transparent about what was interpreted as a causal statement. Previous reviews have identified inconsistent use of causal language in medical journals, see e.g. [7], or use of data-driven variable selection in risk factor analyses [13]. The review was limited to only three high-impact journals, which may prioritise experimental studies over observational studies. Therefore, the sample may not be representative of observational epidemiological studies published in other journals.

Many articles in the review studied a binary outcome and used logistic regression to evaluate the risk factors. The effect estimates, or associations, obtained by taking the exponential of the regression coefficients, were commonly reported as “adjusted odds ratios”. This approach can be useful depending on the purpose. If the conditional odds ratio is the target estimand, the adjusted odds ratio may be a suitable estimator, assuming that the covariates make a sufficient adjustment set and the model is correctly specified. An example of when the conditional odds ratio would be the target estimand is when estimating the odds ratio for an archetypal patient, see e.g. [52].

However, when interest lies in a population effect, a marginal odds ratio is a more relevant estimand. Also, the marginal odds ratio is comparable between studies with different sufficient adjustment sets, making it more interesting from a public health and policy-making perspective. It is well known that, due to non-collapsibility, conditional odds ratios are generally not equal to their marginal counterparts, and the MCOR can not be recovered from the CCOR for a reader without access to the data [41]. It would have been desirable to focus on other aspects in this investigation, but in light of the review’s results, this question has re-emerged.

As illustrated by the simulation study, adjusted estimators of the MCOR are preferable over estimators of the CCOR when the population average causal effect is of interest. As the CCOR is not the same as the MCOR, the bias will not decrease by collecting a larger data set. When marginalising over a sufficient adjustment set of pre-treatment covariates, interpretation is easier in the sense that the resulting marginal OR represent a total causal effect.

The estimators of the MCOR presented here can be implemented using, for example, logistic regression. Given that the model specification is correct, the MCOR can be unbiasedly estimated. In the simulation study, the regression imputation estimators were rather robust, even when the model was misspecified. Estimating the MCOR nonparametrically avoids the dependence on model assumptions, but may call for larger data sets. Models such as random forests are typically data hungry, requiring both many observations and variables. The random forest estimation shows worse performance than the parametric methods. Similar findings were presented in Naimi et al. (2021) [51].

The simulation design was chosen to highlight the findings from the literature review in the familiar structure that brings forth the Table 2 fallacy [42]. Although a single data-generating process was introduced, the simulations present two different scenarios with two possible risk factors included in the same data. The simulation study provides an empirical illustration of an issue already known from theory. Therefore, the points shown through the simulation are general and not specific to the presented data-generating process. However, the size and direction of the bias depend on the data.

The current paper does not focus on unmeasured confounding, a very critical issue in any causal analysis with observational data. Causal graphs are no guarantee against confounding bias, but they do increase the transparency of assumptions made by researchers and force a careful selection of covariates. In addition, they invite an elaborate discussion on the risk of confounding bias. Furthermore, the causal inference literature offers tools for sensitivity analysis that can be combined with the estimators exemplified in the current paper.

## Conclusions

We found that predictive and causal aims are conflated in risk factor analyses. In simulations, we demonstrate how current regression practices may fail to recover a causal effect of a risk factor. Whether the aim is causal or predictive, it should be clearly stated, and analyses and conclusions should be consistent with the stated goal. If the goal is predictive, regression analysis with data-driven variable selection can work well. If the goal is causal, the estimand of interest should be clearly defined, and variables selected based on the underlying causal structure. Our simulations also illustrate that non-parametric methods don’t always outperform parametric ones.

## Supplementary Information

Supplementary Material 1.

## Data Availability

All data in this paper were obtained by simulation. R scripts that generate data, figures, and tables can be downloaded from https://github.com/IngWae/cov_sel_strat. A list of articles included in the literature review is also available at https://github.com/IngWae/cov_sel_strat.

## Abbreviations

AIC: Akaike information criterion
AIPW: Augmented inverse probability weighting
AIPW${}^{*}$: Augmented inverse probability weighting with misspecified propensity score model
ALL: All covariates
BMJ: British Medical Journal
CCOR: Conditional causal odds ratio
DAG: Directed acyclic graph
DMT: Disease-modifying therapy
JAMA: Journal of the American Medical Association
MCOR: Marginal Causal odds ratio
MCSE: Monte Carlo standard error
MS: Multiple Sclerosis
MSE: Mean squared error
NEJM: New England Journal of Medicine
npRI: Non-Parametric Regression imputation
npAIPW: Non-parametric Augmented inverse probability weighting
RI: Regression imputation
SS: Stepwise selection
TTN: Transient Tachypnoea of the newborn
UA: Unadjusted odds ratio
UPF: Univariable pre-filtering
