# Supplementary material for: Covariate selection strategies and estimands - a review of current practice of risk factor analysis from a causal perspective
Source: BMC Med Res Methodol. 2025 Nov 19;25:260. doi: 10.1186/s12874-025-02704-0 (PMC12629056; doi:10.1186/s12874-025-02704-0)
Supplement: Supplementary file 1 — Supplementary Material 1. [file 12874_2025_2704_MOESM1_ESM.pdf]

Appendix A Literature Review

A.1 Search String and List of Articles

The search string used for the literature review:

((regression) AND ((”risk factors”) OR (”risk factor”))) AND (”Lancet Microbe”[Journal] OR ”Lancet Digit Health”[Journal] OR ”Lancet Child Adolesc Health”[Journal] OR ”Lancet Planet Health”[Journal] OR ”Lancet Gastroenterol Hepatol”[Journal] OR ”Lancet Public Health”[Journal] OR ”Lancet Haematol”[Journal] OR ”Lancet HIV”[Journal] OR ”Lancet Psychiatry”[Journal] OR ”Lancet Diabetes Endocrinol”[Journal] OR ”Lancet Glob Health”[Journal] OR ”Lancet Respir Med”[Journal] OR ”Lancet Neurol”[Journal] OR ”Lancet Infect Dis”[Journal] OR ”Lancet Oncol”[Journal] OR ”N Engl J Med”[Journal] OR ”JAMA Netw Open”[Journal] OR ”JAMA Cardiol”[Journal] OR ”JAMA Oncol”[Journal] OR ”JAMA Dermatol”[Journal] OR ”JAMA Intern Med”[Journal] OR ”JAMA Neurol”[Journal] OR ”JAMA Ophthalmol”[Journal] OR ”JAMA Otolaryngol Head Neck Surg”[Journal] OR ”JAMA Pediatr”[Journal] OR ”JAMA Psychiatry”[Journal] OR ”JAMA Surg”[Journal] OR ”JAMA”[Journal] OR ”BMJ Mil Health”[Journal] OR ”BMJ Health Care Inform”[Journal] OR ”BMJ Evid Based Med”[Journal] OR ”BMJ Sex Reprod Health”[Journal] OR ”BMJ Lead”[Journal] OR ”BMJ Open Qual”[Journal] OR ”BMJ Paediatr Open”[Journal] OR ”BMJ Glob Health”[Journal] OR ”BMJ Open Ophthalmol”[Journal] OR ”BMJ Open Gastroenterol”[Journal] OR ”BMJ Open Diabetes Res Care”[Journal] OR ”BMJ Open Respir Res”[Journal] OR ”BMJ Open”[Journal] OR ”BMJ Qual Saf”[Journal] OR ”BMJ Support Palliat Care”[Journal] OR ”BMJ Case Rep”[Journal] OR ”BMJ”[Journal])

The full list of articles is shared here https://github.com/IngWae/cov_sel_strat

A.2 Statistical Guidelines at the Journals

Table A1: Statistical guidelines at the included journals.

Journal	Description
The Lancet	*STROBE statement*
The BMJ	*STROBE statement*<br>*SAMPL guidelines*
JAMA	*Only allow causal language in RCTs*<br>*STROBE statement*<br>*"Reporting statistical information in medical journal articles"*<br>*Clinical, statistical or other rationale for including covaraites should be described*
NEJM	*Do not allow causal language in observational studies where only associations can be estimated*<br>*Show distribution of covariates by treatment level*<br>*Quantify sensitivity to potential confounding bias*

Appendix B Data Learner

Table B2: Variables mimicked from Louapre et al [21].

Variable	Description	Target
Age	Patient's age.	$\bar{x} = 44.6, s = 12.8$ $range = (18, 85)$
Male	1 if patient is male, 0 otherwise.	$\hat{p} = 0.282$
Smoking	1 if patient is a smoker, 0 otherwise.	$\hat{p} = 0.095$
BMI	Patient's BMI.	$range = (14, 46)^*$
Obesity	1 if patient's BMI is 30 or higher, 0 otherwise.	$\hat{p} = 0.069$
Diabetes	1 if patient has diabetes, 0 otherwise.	$\hat{p} = 0.046$
Pulmonary Disease	1 if patient has pulmonary disease, 0 otherwise.	$\hat{p} = 0.043$
Cardiovasular Disease	1 if patient has diabetes, 0 otherwise.	$\hat{p} = 0.066$
Disease Course	Type of Multiple Sclerosis (MS).	
- CIS	1 if patient is diagnosed with Clinically Isolated Syndrome, 0 otherwise.	$\hat{p} = 0.017$
- RRMS	1 if patient is diagnosed with Relapsing-Remitting MS, 0 otherwise.	$\hat{p} = 0.79$
- SPMS	1 if patient is diagnosed with Secondary Progressive MS, 0 otherwise.	$\hat{p} = 0.138$
- PPMS	1 if patient is diagnosed with Primary Progressive MS, 0 otherwise.	$\hat{p} = 0.0489$
EDSS	Expanded Disability Status Scale.	$md = 2$, $range = (0, 9.5)$
DMT	Disease Modifying Therapy.	
- None	1 if patient is not treated with any DMT, 0 otherwise.	$\hat{p} = 0.182$
- Interferon beta	1 if patient is treated with Interferon beta, 0 otherwise.	$\hat{p} = 0.058$
- Glatiramer	1 if patient is treated with Glatiramer, 0 otherwise.	$\hat{p} = 0.095$
- Teriflunomide	1 if patient is treated with Teriflunomide, 0 otherwise.	$\hat{p} = 0.095$
- Dimethylfumarate	1 if patient is treated with Dimethylfumarate, 0 otherwise.	$\hat{p} = 0.101$
- Natalizumab	1 if patient is treated with Natalizumab, 0 otherwise.	$\hat{p} = 0.164$
- Fingolimod	1 if patient is treated with Fingolimod, 0 otherwise.	$\hat{p} = 0.121$
- Ocrelizumab	1 if patient is treated with Ocrelizumab, 0 otherwise.	$\hat{p} = 0.11$
- Rituximab	1 if patient is treated with Rituximab, 0 otherwise.	$\hat{p} = 0.049$
- Cladribine	1 if patient is treated with Cladribine, 0 otherwise.	$\hat{p} = 0.009$
- Alemtuzumab	1 if patient is treated with Alemtuzumab, 0 otherwise.	$\hat{p} = 0.003$
- Other	1 if patient is treated with any another DMT, 0 otherwise.	$\hat{p} = 0.014$
COVID-19 Severity	1 if patient has severe COVID-19, 0 otherwise.	$\hat{p} = 0.21$

**See also Obesity.*

Table B3: Data generating process: Covariates and treatments.

Variable	Model	Dependencies	Parameters
**Age** (X_A)	$\lfloor$Lognormal$\rfloor$		$\mu = \ln(44.6 + 0.55) - \frac{1}{2}\sigma^2,\ \sigma^2 = \ln[1 + (\frac{12.8}{44.6})^2]$
**Male** (X_M)	Bernoulli		$p = \frac{98}{347}$
**Smoking** (X_S)	Logit	X_M, X_A<br>X_A^2, $X_A \cdot X_M$	$\beta_0, \beta = \frac{1}{10}(5\ 0.01\ 0.002\ 0.01)$
**BMI** (X_B)	Shifted χ^2	X_A, X_M,<br>X_S	$k = 0.1 \cdot X_A + 2 \cdot X_M + 6 \cdot X_S -$<br>$0.15 \cdot X_A \cdot X_S - 3 \cdot X_S \cdot X_M$<br>$Shift = c$
**Diabetes** (X_D)	Logit	X_A, X_M,<br>X_S, X_B	$\beta_0, \beta = \frac{1}{10}(0.5\ 1\ 1\ 1)$
**Cardiovascular Disease** (X_{CD})	Logit	X_A, X_M,<br>X_S, X_B,<br>X_D	$\beta_0, \beta = \frac{1}{10}(0.5\ 2\ 4\ 0.5\ 3)$
**Pulmonary Disease** (X_{PD})	Logit	X_A, X_M,<br>X_S, X_B,	$\beta_0, \beta = \frac{1}{10}(0.5\ 0.1\ 5\ 0.1)$
**Disease Course** ($\boldsymbol{X_{DC}}$):<br>*CIS*, RRMS, SPMS, PPMS*	Multinomial Logit	X_A, X_M,<br>X_S, X_D,<br>X_{CD}, X_{PD}	$\beta_0, \beta = \frac{1}{50}\begin{pmatrix} 3 & -1 & 5 & 3 & 3 & 3 & 2 \\ 3 & -1 & 5 & 5 & 5 & 3 & 2 \\ 2 & 2 & 5 & 2 & 2 & 2 & 2 \end{pmatrix}$
**EDSS** (X_E)	Discretized Shifted χ^2	$\boldsymbol{X_{DC}}$	$k = 7$<br>$Shift = \exp(-2 \cdot CIS - 1 \cdot RRMS + 1 \cdot SPMS + 2 \cdot PPMS)$
**Disease Modifying Therapy** ($\boldsymbol{X_{DMT}}$):<br>*None, Interferon Beta, Glatiramer, Natalizumab, Other, Fingolimod, Teriflunomide, Dimethylfumarate, Ocrelizumab, Rituximab, Cladribine, Alemtuzumab**	Multinomial Logit	X_D, X_{CD},<br>X_{PD},$\boldsymbol{X_{DC}}$<br>X_E, X_E^2<br>$X_E \cdot (X_D, X_{CD}, X_{PD})$	$\beta_0, \beta = \begin{pmatrix} 0.7 & 0.7 & 0.7 & 0.5 & 0.5 & -0.2 & 0.1 & -0.1 & 0.1 & 0.01 & -0.001 & 0.001 \\ -0.5 & -0.5 & -0.5 & 0.7 & 0.7 & -0.2 & 0.1 & -0.1 & 0.1 & 0.008 & 0.0011 & 0.0059 \\ -0.5 & -0.5 & -0.5 & 0.4 & 0.4 & -0.2 & 0.1 & 0.3 & -0.01 & 0.006 & 0.0032 & 0.0108 \\ 0.5 & 0.5 & 0.5 & 0.4 & 0.4 & -0.2 & 0.1 & 0.3 & -0.01 & 0.004 & 0.0053 & 0.0157 \\ 0.5 & 0.5 & 0.5 & 0.1 & 0.1 & 0.5 & 0.1 & 0.3 & -0.01 & 0.002 & 0.0074 & 0.0206 \\ -0.1 & -0.1 & -0.1 & 0.3 & 0.3 & 0.5 & 0.1 & 0.3 & -0.01 & 0 & 0.095 & 0.0255 \\ 0.3 & 0.3 & 0.3 & -0.2 & -0.2 & 0.5 & 0.5 & 0.5 & -0.5 & -0.002 & 0.0116 & 0.0304 \\ -0.1 & -0.1 & -0.1 & -0.2 & -0.2 & 0.5 & 0.1 & 0.5 & -0.5 & -0.004 & 0.0137 & 0.0353 \\ -0.1 & -0.1 & -0.1 & -0.2 & -0.2 & 0.5 & 1 & 0.5 & -0.5 & -0.006 & 0.0158 & 0.0402 \\ -0.1 & -0.1 & -0.1 & -0.2 & -0.2 & 0.5 & 0.1 & 0.1 & 0.05 & -0.008 & 0.0179 & 0.0451 \\ 0.5 & 0.5 & 0.5 & -0.2 & -0.2 & 0.5 & 0.1 & 0.1 & 0.05 & -0.01 & 0.02 & 0.05 \end{pmatrix}$
**Risk by DMT** ($\boldsymbol{X_{RISK}}$):			
**No treatment** (X_{NT})	Categorisation of DMT	1 if DMT is None, 0 otherwise.	
**No risk** (X_{NR})	——"——	1 if DMT is Interferon beta or Glatiramer, 0 otherwise.	
**Low risk** (X_{LR})	——"——	1 if DMT is Teriflunomide, Dimethylfumarate, Natalizumab, or Other, 0 otherwise.	
**Moderate to high risk** (X_{MHR})	——"——	1 if DMT is Fingolimod, Ocrelizumab, Rituximab, Cladribine, or Alemtuzumab, 0 otherwise.	

**Reference level.*

Table B4: Data Generating Process: Outcome.

**Scenario**	Outcome Model	Description	Dependencies ($\boldsymbol{X}$)	Outcome	Parameters	Target
**I**	A	Logistic	$X_A, X_M, X_S, X_B, X_D, X_{CD}, X_{PD}, \boldsymbol{X_{DC}}, X_E$	Y_{NT}	$\beta_0, \beta = \frac{1}{5}(0.2\ 1\ 1.6\ 0.05\ 1.5\ 3.5\ 0.001\ 0.25\ 5\ 0.2\ 1\ 1.5)$	$p(Y(0)=1)=0.19$
				Y_{NR}	$\beta_0, \beta = \frac{1}{5}(0.2\ 1\ 1.6\ 0.05\ 1.5\ 3.5\ 0.001\ 0.25\ 5\ 0.2\ 1\ 1.5)$	$p(Y(1)=1)=0.19$
				Y_{LR}	$\beta_0, \beta = \frac{1}{5}(0.2\ 1\ 1.6\ 0.05\ 1.5\ 3.5\ 0.001\ 0.25\ 5\ 0.2\ 1\ 1.5)$	$p(Y(2)=1)=0.20$
				Y_{MHR}	$\beta_0, \beta = \frac{1}{5}(0.2\ 1\ 1.6\ 0.05\ 1.5\ 3.5\ 0.001\ 0.25\ 5\ 0.2\ 1\ 1.5)$	$p(Y(3)=1)=0.25$
	B	Logistic with interaction between treatment and covariates.	$X_{RISK} \cdot (X_A, X_M, X_S, X_B, X_D, X_{CD}, X_{PD}, \boldsymbol{X_{DC}}, X_E)$	Y_{NT}	$\beta_0, \beta = \frac{1}{7}(0.1\ 2\ 0.2\ 0.015\ 2\ 2\ 1.5\ 1\ 5\ 0.5\ 1.5\ 1.75)$	$p(Y(0)=1)=0.19$
				Y_{NR}	$\beta_0, \beta = \frac{1}{5}(0.4\ 3\ 1\ 0.01\ 1.5\ 1\ 1.75\ 0.25\ 5\ 1\ 5\ 1)$	$p(Y(1)=1)=0.19$
				Y_{LR}	$\beta_0, \beta = \frac{1}{3}(0.75\ 1.5\ 0.5\ 0.02\ 1.6\ 0.5\ 0.01\ 1.2\ 1.6\ 5\ 1.7\ 0.75)$	$p(Y(2)=1)=0.20$
				Y_{MHR}	$\beta_0, \beta = \frac{1}{2}(0.2\ 0.01\ 1.6\ 0.025\ 1.5\ 3.5\ 2\ 5\ 0.5\ 0.2\ 1\ 0.85)$	$p(Y(3)=1)=0.25$
	C	Probit with second-order and interaction terms, and interaction between and covariates.	$X_{RISK} \cdot (X_A, ..., X_A \cdot X_M, ..., \boldsymbol{X_{DC}} \cdot X_E,\ X_A^2, X_B^2, X_E^2, X_A^3, X_A^4, \sqrt{X_A})$	Y_{NT}	$\mu_{NT} = Q^*(\boldsymbol{X}^T\beta_{NT}), \sigma_{NT} = SD(\boldsymbol{X}^T\beta_{NT})$ $\beta_{NT} = (\frac{1}{7}(0.1\ 2\ 2\ 0.015\ 2\ 2\ 1.5\ 1\ 5\ 2\ 1.5\ 1.5\ ), \{0.5\}_{j=1}^{72})$	$p(Y(0)=1)=0.19$
				Y_{NR}	$\mu_{NR} = Q^*(\boldsymbol{X}^T\beta_{NR}), \sigma_{NR} = SD(\boldsymbol{X}^T\beta_{NR})$ $\beta_{NR} = (\frac{1}{5}(0.4\ 1\ 1\ 0.01\ 1.75\ 1.5\ 1.8\ 0.25\ 0.5\ 1\ 1\ 1.1), \{0.75\}_{j=1}^{72})$	$p(Y(1)=1)=0.19$
				Y_{LR}	$\mu_{LR} = Q^*(\boldsymbol{X}^T\beta_{LR}), \sigma_{LR} = SD(\boldsymbol{X}^T\beta_{LR})$ $\beta_{LR} = (\frac{1}{3}(0.5\ 1.5\ 0.5\ 0.015\ 1.6\ 2\ 1.7\ 1.2\ 1.6\ 4\ 1.7\ 1.75), \{1\}_{j=1}^{72})$	$p(Y(2)=1)=0.20$
				Y_{MHR}	$\mu_{MHR} = Q^*(\boldsymbol{X}^T\beta_{MHR}), \sigma_{MHR} = SD(\boldsymbol{X}^T\beta_{MHR})$ $\beta_{MHR} = (\frac{1}{2}(0.025\ 1\ 1\ 0.01\ 1.5\ 2\ 1.5\ 1\ 5\ 0.2\ 1\ 1.25), \{1.25\}_{j=1}^{72})$	$p(Y(3)=1)=0.25$
**II**	A	Logistic	$X_A, X_M, X_S, X_B, X_D, X_{CD}, X_{PD}, \boldsymbol{X_{DC}}, X_E$	Y_{NS}	$\beta_0, \beta = \frac{1}{5}(0.5\ 1\ 0.01\ 1.5\ 2\ 1.5\ 0.25\ 0.5\ 1\ 1$ $0.3\ 0.1\ 0.2\ 0.4\ 0.3\ 0.4\ 0.2\ 0.5\ 0.6\ 0.4\ 0.6\ 0.5)$	$p(Y(0)=1)=0.205$
				Y_S	$\beta_0, \beta = \frac{1}{5}(0.5\ 1\ 0.01\ 1.5\ 2\ 1.5\ 0.25\ 0.5\ 1\ 1$ $0.3\ 0.1\ 0.2\ 0.4\ 0.3\ 0.4\ 0.2\ 0.5\ 0.6\ 0.4\ 0.6\ 0.5)$	$p(Y(1)=1)=0.24$
	B	Logistic with interaction between treatment and covariates.	$X_S \cdot (X_A, X_M, X_S, X_B, X_D, X_{CD}, X_{PD}, \boldsymbol{X_{DC}}, X_E)$	Y_{NS}	$\beta_0, \beta = \frac{1}{3}(0.2\ 1\ 0.01\ 1.5\ 2\ 1.5\ 0.25\ 0.5\ 1.2\ 1$ $0.3\ 0.1\ 0.2\ 0.7\ 0.5\ 0.4\ 0.2\ 0.5\ 0.6\ 0.4\ 0.6\ 0.5)$	$p(Y(0)=1)=0.205$
				Y_S	$\beta_0, \beta = \frac{1}{3}(0.5\ 1.5\ 0.05\ 1.4\ 1.45\ 1.7\ 0.5\ 1.6$ $2.1\ 1.25\ 0.8\ 0.2\ 0.5\ 0.5\ 0.2\ 0.6\ 0.4\ 0.65\ 0.5\ 0.5\ 0.3\ 0.4)$	$p(Y(1)=1)=0.24$
	C	Probit with second-order and interaction terms, and interaction between and covariates.	$X_S \cdot (X_A, ..., X_{DMT}, X_A \cdot X_M, ..., \boldsymbol{X_{DC}} \cdot X_E,\ X_A^2, X_B^2, X_E^2, X_A^3, X_A^4, \sqrt{X_A}))$	Y_{NS}	$\mu_{NS} = Q^*(\boldsymbol{X}^T\beta_{NS}), \sigma_{NS} = SD(\boldsymbol{X}^T\beta_{NS})$ $\beta_{NS} = \frac{1}{5}(0.2\ 1\ 0.01\ 1.5\ 2\ 1.5\ 0.2\ 0.5\ 1\ 1\ 2\ 0.1\ 0.2\ 0.4\ 0.3\ 0.4$ $0.2\ 0.5\ 0.6\ 0.4\ 0.6\ 0.5), \{1\}_{j=1}^{237})$	$p(Y(0)=1)=0.205$
				Y_S	$\mu_S = Q^*(\boldsymbol{X}^T\beta_S), \sigma_S = SD(\boldsymbol{X}^T\beta_S)$ $\beta_S = \frac{1}{5}(0.5\ 1.5\ 0.06\ 1.4\ 1.2\ 1.7\ 0.5\ 1.6\ 2\ 1.25\ 2.7\ 0.2\ 0.5\ 0.5\ 0.2$ $0.6\ 0.2\ 0.65\ 0.5\ 0.5\ 0.3\ 0.4), \{1.5\}_{j=1}^{237})$	$p(Y(1)=1)=0.24$

* *The quantile that yields the desired prevalence listed in Target column.*

Appendix C Overlap

C.1 Scenario I

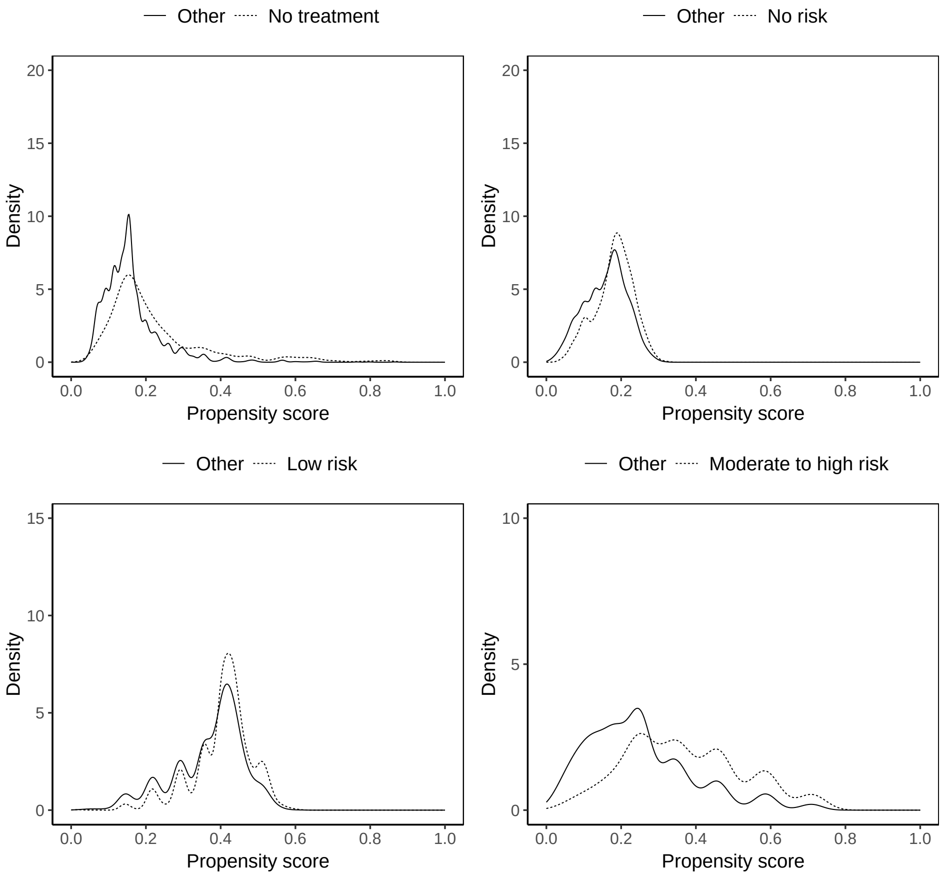

Fig. C1: Overlap in sample 1 under correct model.

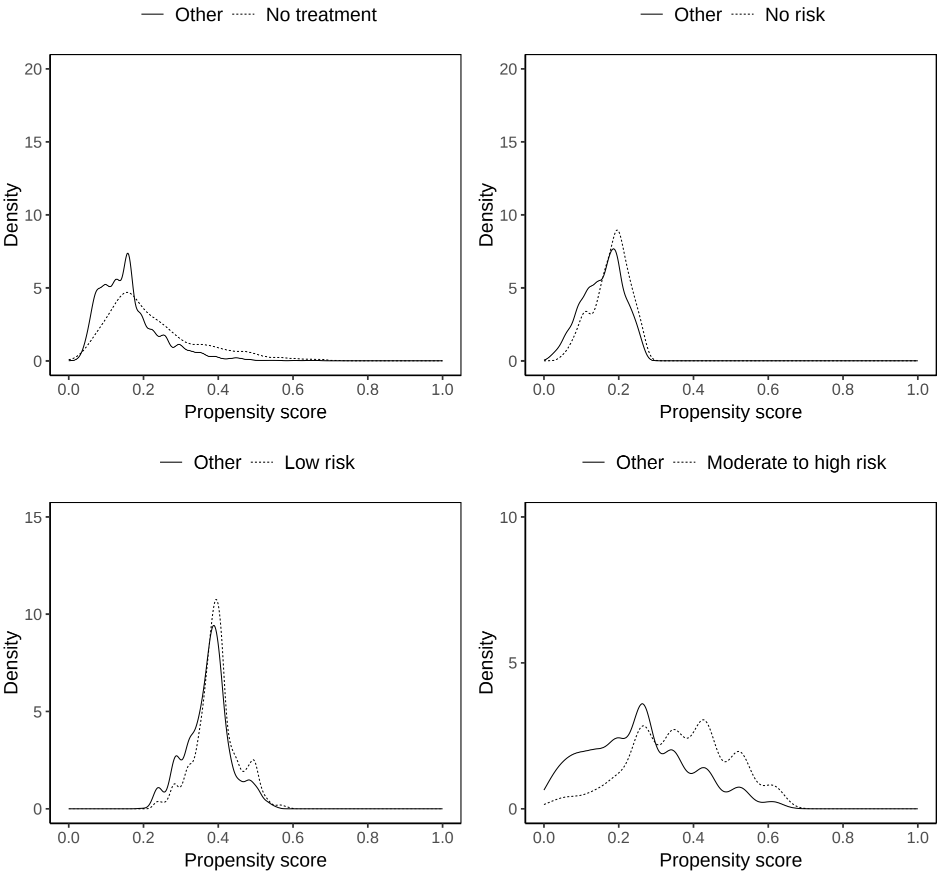

Fig. C2: Overlap in sample 1 under incorrect model.

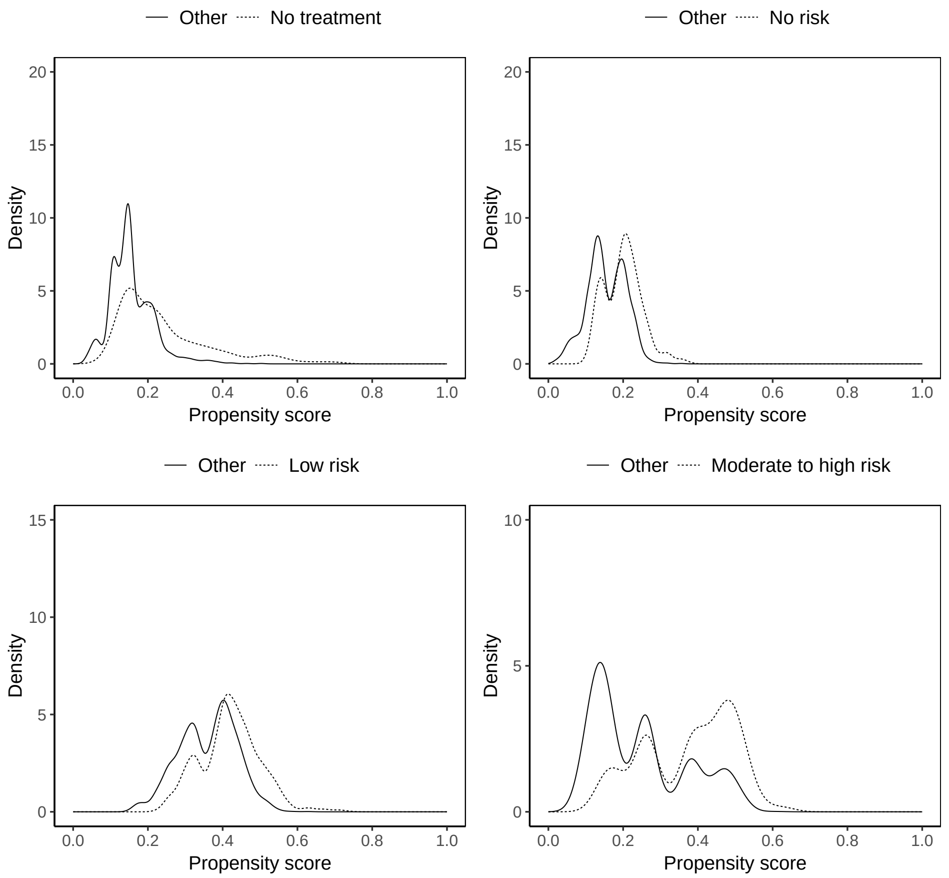

Fig. C3: Overlap in sample 1 under nonparametric model.

C.2 Scenario II

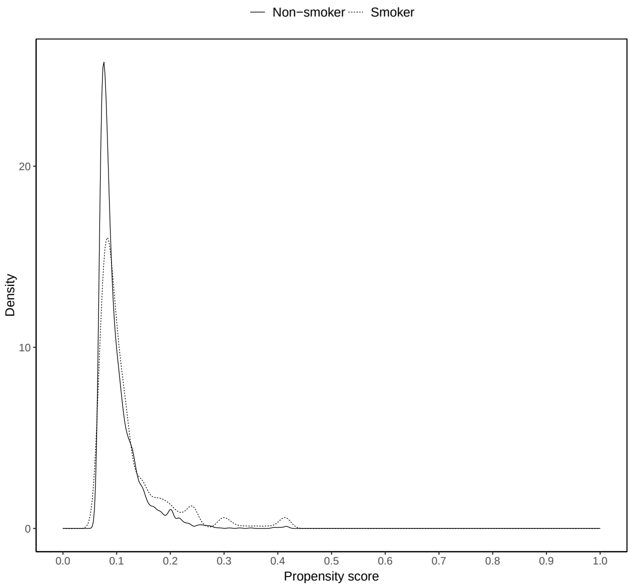

Fig. C4: Overlap in sample 1 under correct model.

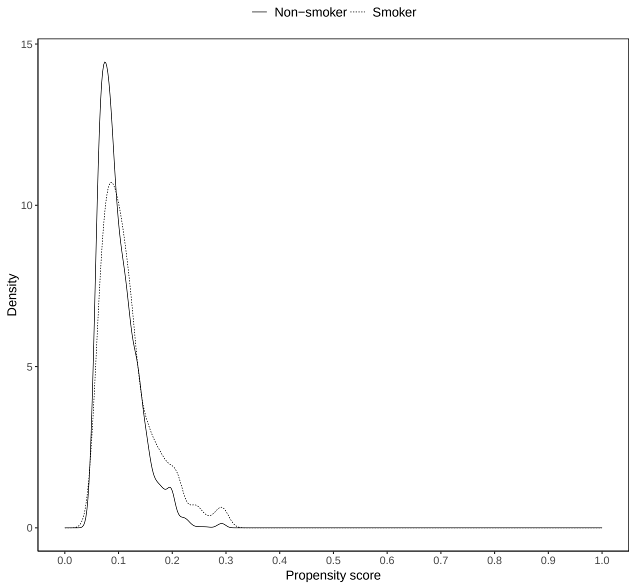

Fig. C5: Overlap in sample 1 under incorrect model.

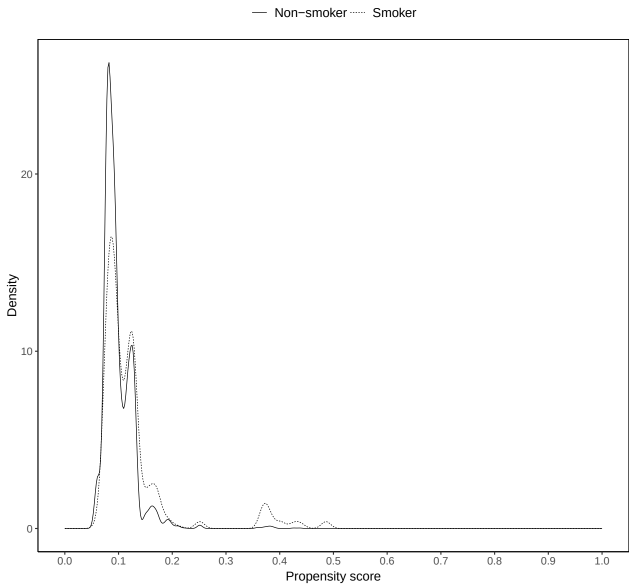

Fig. C6: Overlap in sample 1 under nonparametric model.

Appendix D Monte Carlo Standard Errors

D.1 Scenario I

Table D5: Monte Carlo standard errors of bias and MSE in Scenario I.

	Bias			MSE		
Outcome Model:	A	B	C	A	B	C
$\theta_{1\|0}$						
UA	0.12	0.12	0.12	0.04	0.04	0.04
ALL	0.16	0.22	0.18	0.04	0.11	0.06
SS	0.16	0.22	0.18	0.04	0.11	0.05
UPF	0.16	0.22	0.18	0.04	0.11	0.06
RI	0.16	0.16	0.14	0.04	0.04	0.03
AIPW	0.16	0.16	0.14	0.04	0.04	0.03
AIPW*	0.16	0.16	0.14	0.04	0.05	0.04
npRI	0.14	0.14	0.13	0.03	0.03	0.03
npAIPW	0.15	0.15	0.13	0.03	0.04	0.03
$\theta_{2\|0}$						
UA	0.11	0.11	0.12	0.04	0.03	0.02
ALL	0.14	0.19	0.16	0.03	0.08	0.05
SS	0.14	0.19	0.16	0.03	0.08	0.04
UPF	0.14	0.19	0.16	0.03	0.08	0.04
RI	0.13	0.13	0.12	0.03	0.03	0.02
AIPW	0.13	0.13	0.12	0.03	0.03	0.02
AIPW*	0.13	0.13	0.12	0.03	0.03	0.02
npRI	0.12	0.12	0.12	0.02	0.02	0.02
npAIPW	0.13	0.12	0.12	0.02	0.02	0.02
$\theta_{3\|0}$						
UA	0.12	0.12	0.16	0.11	0.11	0.05
ALL	0.21	0.27	0.26	0.08	0.14	0.19
SS	0.21	0.27	0.25	0.08	0.14	0.18
UPF	0.21	0.27	0.25	0.08	0.14	0.17
RI	0.19	0.19	0.17	0.06	0.06	0.04
AIPW	0.20	0.20	0.18	0.07	0.06	0.05
AIPW*	0.28	0.27	0.24	0.14	0.12	0.16
npRI	0.17	0.16	0.16	0.05	0.05	0.04
npAIPW	0.18	0.17	0.17	0.05	0.05	0.05

D.2 Scenario II

Table D6: Monte Carlo standard errors of bias and MSE in Scenario II.

	Bias			MSE		
Outcome Model:	A	B	C	A	B	C
$\theta_{1\|0}$						
UA	0.23	0.23	0.23	0.28	0.33	0.28
ALL	0.22	0.19	0.22	0.10	0.10	0.11
SS	0.22	0.19	0.22	0.10	0.10	0.10
UPF	0.22	0.19	0.22	0.11	0.10	0.10
RI	0.15	0.13	0.15	0.04	0.02	0.04
AIPW	0.15	0.13	0.15	0.04	0.02	0.03
AIPW*	0.15	0.13	0.15	0.04	0.02	0.04
npRI	0.17	0.15	0.16	0.07	0.06	0.07
npAIPW	0.14	0.11	0.13	0.03	0.02	0.03

Appendix E Bias Under Model Misspecification

To specify a framework for model misspecification of the outcome model note that we assume a model for the observed data

$$P(Y = 1|\boldsymbol{X}, T, \boldsymbol{\beta}) = g_t(\boldsymbol{X}, T, Y, \boldsymbol{\beta}).$$

for some vector of parameters β. Under regularity conditions for the function $g_t()$ and convergence of misspecified regression model parameters, $\hat{\boldsymbol{\beta}} \xrightarrow{p} \boldsymbol{\beta}^*$, we can apply a weak law of large numbers for averages of estimated parameters yielding an asymptotic bias under model misspecification:

$$\frac{E[g_t(\boldsymbol{X}, T, Y, \beta^*)]/1 - E[g_t(\boldsymbol{X}, T, Y, \beta^*)]}{E[g_t'(\boldsymbol{X}, T, Y, \beta^*)]/1 - E[g_t'(\boldsymbol{X}, T, Y, \beta^*)]} - \theta_{t|t'}. \tag{E1}$$

For the Scenarios I and II in the simulation study the asymptotic bias is approximated by drawing a large sample of 1 million observations from the data learner. Estimates of the odds ratios and numerical approximations of the asymptotic bias in Scenario I are presented in Table E7 and in Table E8 for Scenario II.

Table E7: Numerical approximations of the asymptotic bias in samples with n = 1 million. The true MCORs are $\theta_{1|0} = 1$, $\theta_{2|0} = 1.07$, and $\theta_{3|0} = 1.42$.

	Bias		
Outcome Model:	A	B	C
$\theta_{1\|0}$			
UA	-0.13	-0.14	-0.18
ALL	0.01	0.13	-0.01
RI	0.01	0.02	0.00
AIPW	0.01	0.01	0.00
AIPW*	0.00	0.01	0.00
$\theta_{2\|0}$			
UA	-0.14	-0.07	-0.10
ALL	0.01	0.09	0.02
RI	0.00	0.01	0.00
AIPW	0.00	0.01	0.00
AIPW*	0.00	0.01	0.00
$\theta_{3\|0}$			
UA	-0.36	-0.33	-0.16
ALL	0.05	0.15	0.22
RI	0.00	0.01	0.00
AIPW	0.00	0.00	0.00
AIPW*	0.00	0.00	0.00

Table E8: Numerical approximations of the asymptotic bias in samples with n = 1 million. The true MCOR is $\theta_{1|0} = 1.225$.

	Bias		
Outcome Model:	A	B	C
$\theta_{1\|0}$			
UA	0.55	0.67	0.54
ALL	0.09	0.20	0.09
RI	0.01	-0.01	-0.01
AIPW	0.01	-0.01	-0.02
AIPW*	0.01	-0.01	0.00
